# A Critical Analysis of Chemical and Electrochemical Oxidation Mechanisms in Li-Ion Batteries

**DOI:** 10.1021/acs.jpclett.3c03279

**Published:** 2024-01-04

**Authors:** Evan Walter Clark Spotte-Smith, Sudarshan Vijay, Thea Bee Petrocelli, Bernardine L. D. Rinkel, Bryan D. McCloskey, Kristin A. Persson

**Affiliations:** † Department of Materials Science and Engineering, University of California, Berkeley, 210 Hearst Memorial Mining Building, Berkeley, California 94720, United States; ‡ Materials Science Division, Lawrence Berkeley National Laboratory, 1 Cyclotron Road, Berkeley, California 94720, United States; § Department of Chemical and Biomolecular Engineering, University of California, Berkeley, 201 Gilman Hall, Berkeley, California 94720, United States; ∥ Energy Storage and Distributed Resources, Lawrence Berkeley National Laboratory, 1 Cyclotron Road, Berkeley, California 94720, United States; ⊥ Molecular Foundry, Lawrence Berkeley National Laboratory, 1 Cyclotron Road, Berkeley, California 94720, United States

## Abstract

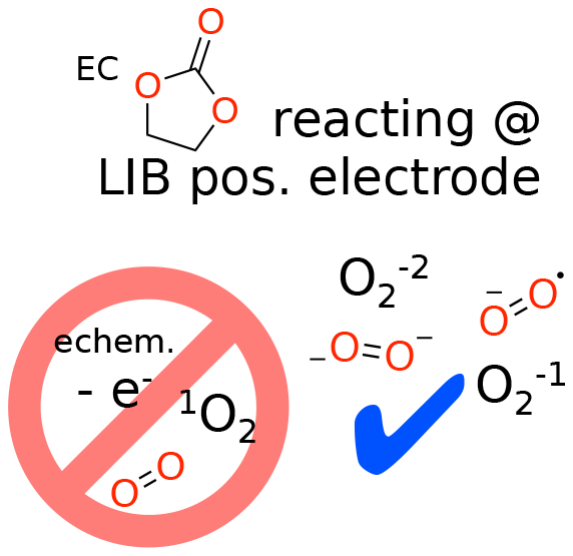

Electrolyte decomposition limits the lifetime of commercial lithium-ion batteries (LIBs) and slows the adoption of next-generation energy storage technologies. A fundamental understanding of electrolyte degradation is critical to rationally design stable and energy-dense LIBs. To date, most explanations for electrolyte decomposition at LIB positive electrodes have relied on ethylene carbonate (EC) being chemically oxidized by evolved singlet oxygen (^1^O_2_) or electrochemically oxidized. In this work, we apply density functional theory to assess the feasibility of these mechanisms. We find that electrochemical oxidation is unfavorable at any potential reached during normal LIB operation, and we predict that previously reported reactions between the EC and ^1^O_2_ are kinetically limited at room temperature. Our calculations suggest an alternative mechanism in which EC reacts with superoxide (O_2_^–^) and/or peroxide (O_2_^2–^) anions. This work provides a new perspective on LIB electrolyte decomposition and motivates further studies to understand the reactivity at positive electrodes.

Electrolyte design is one of the most significant remaining challenges in the development of lithium-ion battery (LIB) technologies. To be practically useful, an electrolyte must simultaneously possess a number of key properties, including high Li^+^ conductivity and transference number, low viscosity, and compatibility with the battery’s positive and negative electrodes.^1^ The latter requirement, that electrolytes must be stable at both electrodes, is especially challenging. To achieve a high energy density, LIBs operate at extreme potentials, as low as 0.1 V for common graphitic negative electrodes (in this work, all potentials are referenced to the reduction potential of Li^+^) and, depending on the composition of the positive electrode, as high as 4.2–4.5 V for lithium nickel manganese cobalt oxides (NMC)^2^ or 4.5–4.8 V for novel disordered rock salts (DRX).^3^ In the reactive environment created at these potentials, most electrolytes tend to degrade over time, leading to decreased Coulombic efficiency and irreversible capacity loss.^4^ To continue to improve the lifetime of LIBs and to enable the deployment of next-generation electrodes like DRX, it is essential either to prevent electrolyte decomposition reactions entirely or to promote the formation of passivation films, known as solid–electrolyte interphases (SEIs) when formed on the negative electrode^5^ and cathode–electrolyte interphases (CEIs) when formed on the positive electrode.^6^

Commercial LIB electrolytes are solutions composed of lithium hexafluorophosphate (LiPF_6_) dissolved in a mixture of organic carbonates, such as ethylene carbonate (EC) and ethyl methyl carbonate.^7,8^ For many years, SEI formation mechanisms for carbonate/LiPF_6_ electrolytes at LIB negative electrodes have been studied in detail; experimentally observed products have been mapped to reactants through a combination of experimental characterization techniques,^9,10^ atomistic modeling,^11−13^ and chemical reaction network (CRN) analysis.^14,15^ By comparison, degradation mechanisms at positive electrodes have received less attention and are less well understood.

Electrochemical oxidation and chemical oxidation have been proposed in the literature as explanations for EC decomposition at potentials of ≳4 V^16,17^ (in general, exact onset potentials for electrolyte decomposition and other degradation processes depend on the electrode composition, among other factors).^18^ In electrochemical oxidation, EC loses an electron to the electrode, after which it can react with other salt and solvent molecules.^19^ Chemical oxidation does not involve direct electron transfer between the electrode and electrolyte but instead involves the attack by so-called “reactive oxygen”. Diatomic oxygen (O_2_) has been detected evolving at high potentials from transition metal oxide positive electrodes, including NMC (∼4.2 V)^20,21^ and DRX (∼4.5 V).^22,23^ Some reports studying NMC materials claim that this O_2_ is released in the singlet excited state (^1^O_2_),^10,24−27^ which is considered to be more reactive than the triplet ground state (^3^O_2_).^28,29^

In this work, we evaluate both of these proposed reaction mechanisms using density functional theory (DFT) calculations. We show that electrochemical oxidation of EC is unlikely to occur at typical operating voltages in LIBs and that chemical oxidation by singlet oxygen is potentially also infeasible due to sluggish kinetics. These findings suggest that the potential-dependent degradation of EC at LIB positive electrodes occurs via alternate mechanisms. We conclude by hypothesizing a decomposition route that requires neither electrochemical oxidation nor reactions with ^1^O_2_. Specifically, on the basis of the observations of partial oxygen redox to peroxide (O_2_^2–^) or “peroxo-like” species in transition metal oxide electrodes and reports of reactions between organic carbonates and superoxide anions (O_2_^–^), we suggest that oxygen anions may favorably react with and degrade EC.

We begin by considering electrochemical oxidation. The hypothesis that EC reacts electrochemically at battery positive electrodes appears suspect on the basis of the available experimental literature. Reported oxidation potentials for EC vary, but if we limit our consideration to measurements made using nonreactive electrodes such as platinum or glassy carbon, aiming to eliminate the possibility of reactions between EC and electrode surfaces, the reported values are between 4.8 and 6.5 V.^16,30−34^ Even the low end of this range is considerably higher than typical LIB operating potentials. However, Chen^35^ recently suggested that concentration effects may decrease the effective oxidation potential of EC. That is, if the products of electrochemical oxidation are sufficiently short-lived or in sufficiently low concentration at steady state, the reaction could become favorable at potentials below EC’s standard oxidation potential.

To evaluate the possibility of electrochemical EC oxidation, we consider the elementary steps of charge transfer to EC. In the simplest mechanism, EC is oxidized as
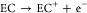
1where e^–^ is an electron and EC^+^ is the oxidized form of EC (see Figure 1a). Our computations (see the Supporting Information for computational methods) show that this reaction has a standard oxidation potential *E*° of 6.98 V, which is significantly higher than the experimentally measured range.

**Figure 1:**
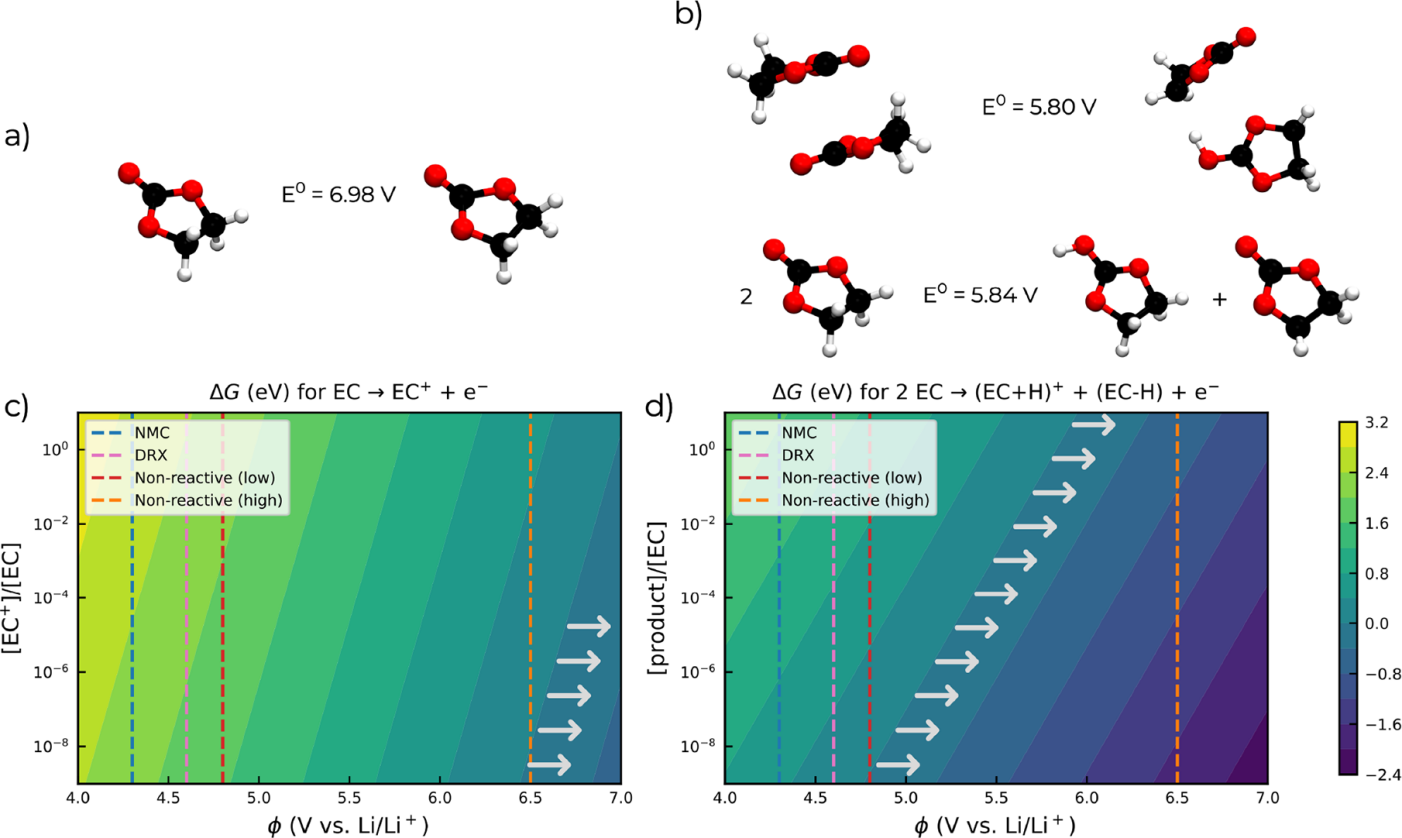
(a) Depiction of the oxidation reaction EC → EC^+^ + e^–^ (reaction 1), with three-dimensional (3D) structures for EC and EC^+^. (b) Depictions of the concerted dissociative oxidation reaction 2EC → (EC+H)^+^ + (EC–H) + e^–^ (reaction 2), with 3D structures of the reactants and products as clusters (top) and isolated molecules (bottom). (c) Free energy change Δ*G* for the stepwise electrochemical oxidation of EC (reaction 1), as a function of potential and relative concentration. (d) Δ*G* for the concerted dissociative oxidation of EC (Reaction 2) as a function of potential and relative concentration, where we assume that (EC+H)^+^ and EC–H are at the same concentration, denoted as [product]. Vertical lines in panels c and d indicate typical maximum potentials, for example, NMC (blue, shown at 4.3 V) and DRX (pink, shown at 4.6 V) positive electrodes, as well as the range of reported electrochemical oxidation potentials of EC at nonreactive electrodes (red and orange). For all points to the right of the arrow tails (at the Δ*G* = 0.0 eV line), electrochemical EC oxidation is predicted to be thermodynamically accessible.

The mechanism of EC oxidation may change when additional solvent molecules are involved. Xing and Borodin^19^ previously applied DFT and Møller–Plesset perturbation theory to study the oxidation of a cluster of two EC molecules, EC_2_. They found that, during the optimization of the oxidized cluster EC_2_^+^, a proton transferred from one EC to the other, making the reaction overall
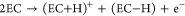
2This spontaneous transfer of a proton implies that the initial elementary step of electrochemical EC oxidation is a concerted reaction involving both charge transfer and proton transfer. We confirm that during DFT optimization of EC_2_^+^, proton transfer occurs, though this does not necessarily imply that EC oxidation is concerted. The calculated standard oxidation potential for reaction 2 is *E*° = 5.80 V using the free energies of the reactant and product clusters (Figure 1b, top) and *E*° = 5.84 V using reactants and products at infinite separation (Figure 1b, bottom), within the experimentally measured range at nonreactive electrodes.

Especially in the condensed phase, it can be difficult to determine if electron transfer reactions are stepwise (with charge transfer followed by additional bond cleavage and formation steps) or concerted.^36^ Even if intermediates form following charge transfer in a stepwise mechanism, those intermediates may be too short-lived or at overly low concentrations to be experimentally observed. Although the calculated standard oxidation potential of the concerted dissociative oxidation given by reaction 2 is in better agreement with experimental characterization compared to that of reaction 1, we cannot at present say with certainty if electrochemical EC oxidation follows a stepwise or concerted pathway. This is especially true given Chen’s argument about concentration effects. Given this uncertainty, we must consider both stepwise and concerted dissociative electrochemical EC oxidation.

To determine whether electrochemical oxidation is feasible under battery operating conditions, we compute the free energy change (Δ*G*) as
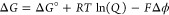
3where Δ*G*° is the free energy change under standard temperature, pressure, and concentration conditions at a fixed standard potential ϕ_0_, *R* is the ideal gas constant, *T* is the absolute temperature in kelvin, *Q* is the reaction quotient, which is
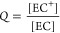
4for the stepwise oxidation given by reaction 1 and
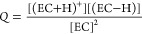
5for the concerted dissociative oxidation given by reaction 2, *F* is Faraday’s constant, and Δϕ = ϕ – ϕ_0_ is the difference between the potential at the positive electrode and the reference potential. Here, we use the vacuum potential of the electron as ϕ_0_.^37^

Neither the stepwise nor the concerted dissociative electrochemical oxidation reactions are feasible under the standard conditions, with standard oxidation potentials being significantly higher than 5 V. However, as indicated in eq 3, the feasibility of reactions 1 and 2 depends on both operating potential ϕ and the relative steady-state concentrations of the oxidation products.

Figure 1 shows Δ*G* for different ϕ values (*x*-axis) and relative concentrations (*y*-axis) for the stepwise (c) and concerted (d) mechanisms. The lower the relative concentration (along the *y*-axis for a fixed *x*-axis value), the greater the favorability of the reaction. Likewise, the reaction is more favorable as the potential is increased (along the *x*-axis for a fixed *y*-axis value).

At all relative concentrations considered (as low as 10^–9^), we predict that a potential of >6 V would need to be applied for reaction 1 to be thermodynamically favorable. Even considering possible error in our DFT calculations, this strongly suggests that the stepwise electrochemical oxidation of EC is not the dominant mechanism of EC degradation at LIB positive electrodes during normal battery operation. Because the standard oxidation potential of reaction 2 is considerably lower than that of reaction 1, concerted dissociative oxidation is more favorable at all potentials considered. The effect of relative concentration is also more significant in reaction 2, which involves two products (see eq 5). As such, Figure 1d shows that it could be possible for concerted dissociative EC oxidation to occur at applied potentials as low as ∼4.8 V. If the concerted dissociative mechanism is possible in real LIB electrolytes and the steady-state product concentrations are extremely low, it may be possible for electrochemical EC oxidation to occur at some high-voltage positive electrodes. However, even in this case, we predict that electrochemical oxidation cannot occur at lower potentials used with, e.g., NMC electrodes.

As we predicted that electrochemical oxidation of EC is unlikely to occur at applied potentials relevant to LIB operation, we now study the feasibility of chemical oxidation by ^1^O_2_. We compute the free energies and free energy barriers for reaction mechanisms previously proposed in the literature, all taken at room temperature (298.15 K).

We identified two elementary mechanisms for chemical reactions between ^1^O_2_ and EC (Figure 2). In the first (red) pathway, originally suggested by Jung et al.,^24^^1^O_2_ initially reacts with EC to form water and 1,3-dioxolane-2,4-dione, a dicarbonyl species. The second (yellow) pathway, proposed by Freiberg et al.,^38^ results in the formation of H_2_O_2_ and vinylene carbonate (VC).

**Figure 2:**
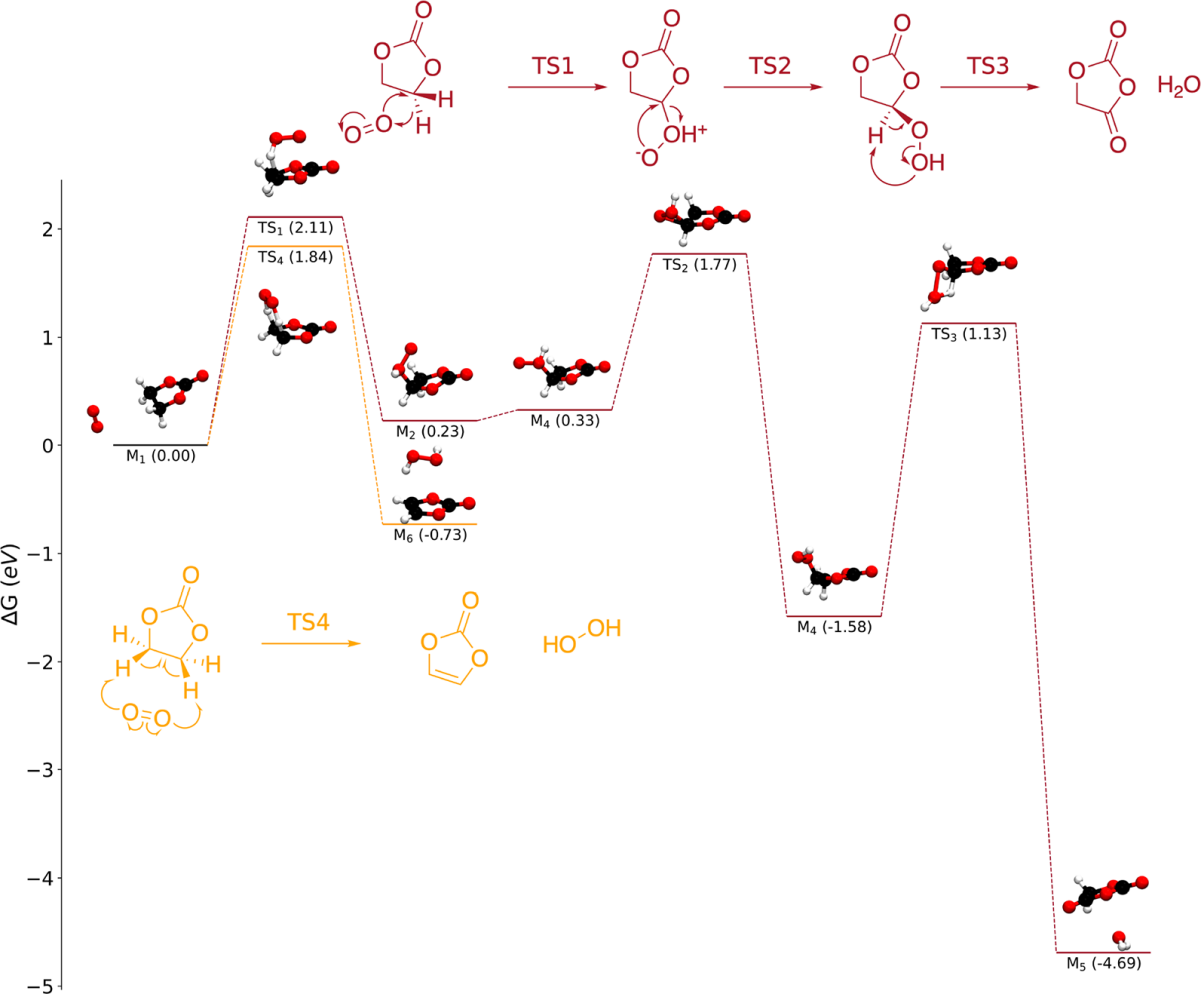
Energy diagrams for two reactions between ^1^O_2_ and EC: a multistep reaction that eventually forms H_2_O and a dicarbonyl species (red) and one in which ^1^O_2_ abstracts two hydrogen atoms from EC to form H_2_O_2_ and vinylene carbonate (VC) in a single concerted step (yellow). Both pathways are thermodynamically favorable but severely kinetically limited.

The water-forming pathway begins (M_1_ → M_2_; Δ*G*^⧧^ = 2.11 eV) with ^1^O_2_ abstracting a hydrogen atom and attaching to the EC in a concerted reaction. The result of this attachment is the zwitterionic complex M_2_. After rotation (M_2_ → M_3_; Δ*G* = 0.10 eV), a rearrangement occurs (M_3_ → M_4_; Δ*G*^⧧^ = 1.44 eV), replacing the zwitterionic −OHO group with a hydroperoxide group (−OOH). We note that we considered a direct reaction between M_1_ and M_4_, but all attempts to locate a transition state for the M_1_ → M_4_ reaction resulted in optimizing the M_1_ → M_2_ or M_3_ → M_4_ transition state. In the final step (M_4_ → M_5_; Δ*G*^⧧^ = 2.71 eV), water is eliminated, leaving a carbonyl group. While this pathway is overall thermodynamically favorable (M_1_ → M_5_; Δ*G* = −4.69 eV), it is severely kinetically limited, with all three free energy barriers being >1.0 eV and two of the three being >2.0 eV. To exemplify the sluggish kinetics, consider the M_1_ → M_2_ reaction proceeding at room temperature in a pure EC solution (concentration of ≈15 M) with a dissolved ^1^O_2_ concentration of 1.56 mM (the solubility of O_2_ in EC is discussed in the Supporting Information). Calculating the rate as
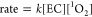
6where *k* is the rate coefficient of the reaction, determined using the Eyring equation
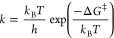
7we predict that the initial rate for this reaction (rate_0_) would be 3.12 × 10^–25^ M s^–1^, which is vanishingly small.

We note that previous studies have suggested that EC chemical oxidation does not end with elimination of a single H_2_O molecule. Following this initial chemical oxidation, Jung et al. suggested that further oxidation by ^1^O_2_ could result in the evolution of CO_2_ and CO,^24^ while Rinkel et al. proposed an alternative pathway to CO_2_ and glycolic acid.^10^ However, given that even the first step of the reaction between EC and ^1^O_2_ is highly unlikely to occur, we have chosen not to pursue these downstream reaction pathways.

Compared to the water-forming pathway, the H_2_O_2_-forming pathway is more straightforward. In a single, concerted step, ^1^O_2_ abstracts two hydrogen atoms from the ethylene carbons in the EC (M_1_ → M_6_; Δ*G*^⧧^ = 1.84 eV), yielding H_2_O_2_ and vinylene carbonate (VC). This mechanism is also thermodynamically favorable, with a Δ*G* of −0.73 eV, but due to the high barrier, we do not expect it to occur appreciably at room temperature (rate_0_ = 1.15 × 10^–20^ M s^–1^). We note that previous computational studies have indicated that the EC + ^1^O_2_ → H_2_O_2_ + VC reaction is kinetically limited. Using the complete active space second-order perturbation theory (CASPT2) multireference method with ANO-L-VDZP basis set in vacuum with the zero-point energy obtained from DFT, Freiberg et al.^38^ predicted a reaction energy barrier (Δ*E*^⧧^) of 1.27 eV. Although we cannot directly compare our predictions with these values, as we performed our calculations in an implicit solvent medium and included enthalpic and entropic terms to calculate a free energy barrier, we can nonetheless say that our prediction agrees qualitatively with the result of Freiberg et al. in that they are significantly larger than what would be expected for a fast reaction.

We have not exhaustively considered all possible reactions between EC and ^1^O_2_, but these findings suggest that ^1^O_2_ may not be as reactive in the presence of EC as had previously been believed. While potentially surprising, the notion that reactions between EC and ^1^O_2_ are kinetically limited appears to be consistent with the most direct evidence of such reactions. Freiberg et al.^38^ fully saturated a solution of EC with O_2_ and illuminated rose Bengal, a salt that can photochemically excite ^3^O_2_ to ^1^O_2_, continuously for 1 h to generate ^1^O_2_ to react with EC. Using online electrochemical mass spectroscopy, they observed that some O_2_ was consumed during the illumination of rose Bengal, on the order of tens of nanomoles for a milliliter sample of EC. They also observed the formation of H_2_O_2_ through colorimetry. Notably, the experiments of Freiberg et al. were performed at 45 °C, which should accelerate the reactions between EC and photogenerated ^1^O_2_; despite this, the reaction appears to be slow.

More recently, Rinkel et al. performed similar experiments using O_2_-saturated EC solutions and rose Bengal that were designed to promote chemical oxidation of EC.^27^ After illumination for 2 h, relatively little reactivity between EC and ^1^O_2_ was observed. Using solution-phase nuclear magnetic resonance (NMR) spectroscopy, the authors found that the amount of water in the solution increased by a factor of ∼3, which indicates some reactions took place in the saturated solution. However, the authors began with an electrolyte with a water content of <10 ppm, which means that the absolute quantity of water formed was minimal. Moreover, Rinkel et al. were unable to detect other expected reaction products, such as vinylene carbonate (VC) and hydrogen peroxide (H_2_O_2_), though as Freiberg et al. note,^38^ even if VC formed, it may be difficult to detect by NMR due to its low concentration and relative instability. In summary, reactions between ^1^O_2_ and EC probably occur to some degree, but we believe that they are not the major drivers of electrolyte decomposition at LIB positive electrodes.

As our calculations call into question the electrochemical as well as chemical oxidation of EC at LIB positive electrodes, we find it necessary to consider alternative mechanisms for the observed reactivity of EC at increased potentials. We propose one such alternative, that EC may indeed combine with “reactive oxygen”, but that the “reactive oxygen” is comprised of oxygen anions superoxide (O_2_^–^) and/or peroxide (O_2_^2–^), rather than ^1^O_2_. This explanation is consistent with the existing literature on oxygen in LIB positive electrodes. It is well-known that oxygen redox occurs within many oxide positive electrodes during battery charging and discharging.^39−43^ Both experimental and theoretical studies have observed peroxides or “peroxo-like” oxygen dimers in the electrode bulk.^44−46^ These dimers have been suggested as intermediates that can eventually lead to neutral O_2_ evolution. More recently, Genreith-Schriever et al.^47^ performed *ab initio* molecular dynamics simulations on LiNiO_2_ electrodes and showed that peroxide dimers form on the electrode surface, which is more directly related to oxygen loss and reactions with electrolyte molecules. Moreover, superoxide species are believed to be important intermediates in metal–air batteries, where they have been linked to the decomposition of carbonate solvents.^48−50^

As with ^1^O_2_, we identified a number of elementary reaction mechanisms between EC and either O_2_^–^ (Figure 3) or O_2_^2–^ (Figure 4) using DFT. It is worth noting that these mechanisms are only suggestions of the initial steps of EC decomposition, and further work must be done to examine how these reactions may lead to more stable decomposition products.

**Figure 3:**
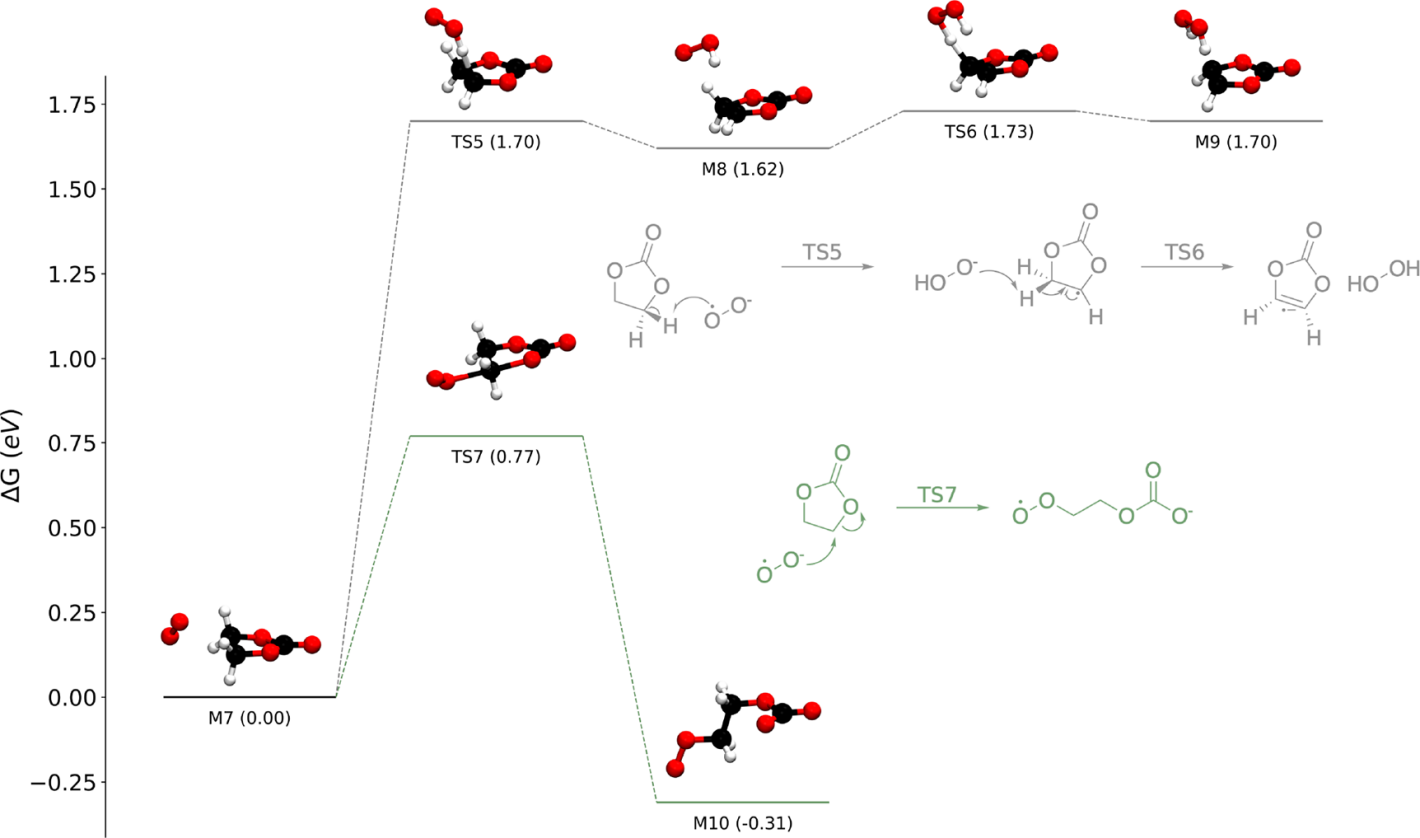
Energy diagrams for reactions between EC and superoxide (O_2_^–^), including a route forming H_2_O_2_ and reduced VC (gray) and a nucleophilic substitution (green).

**Figure 4:**
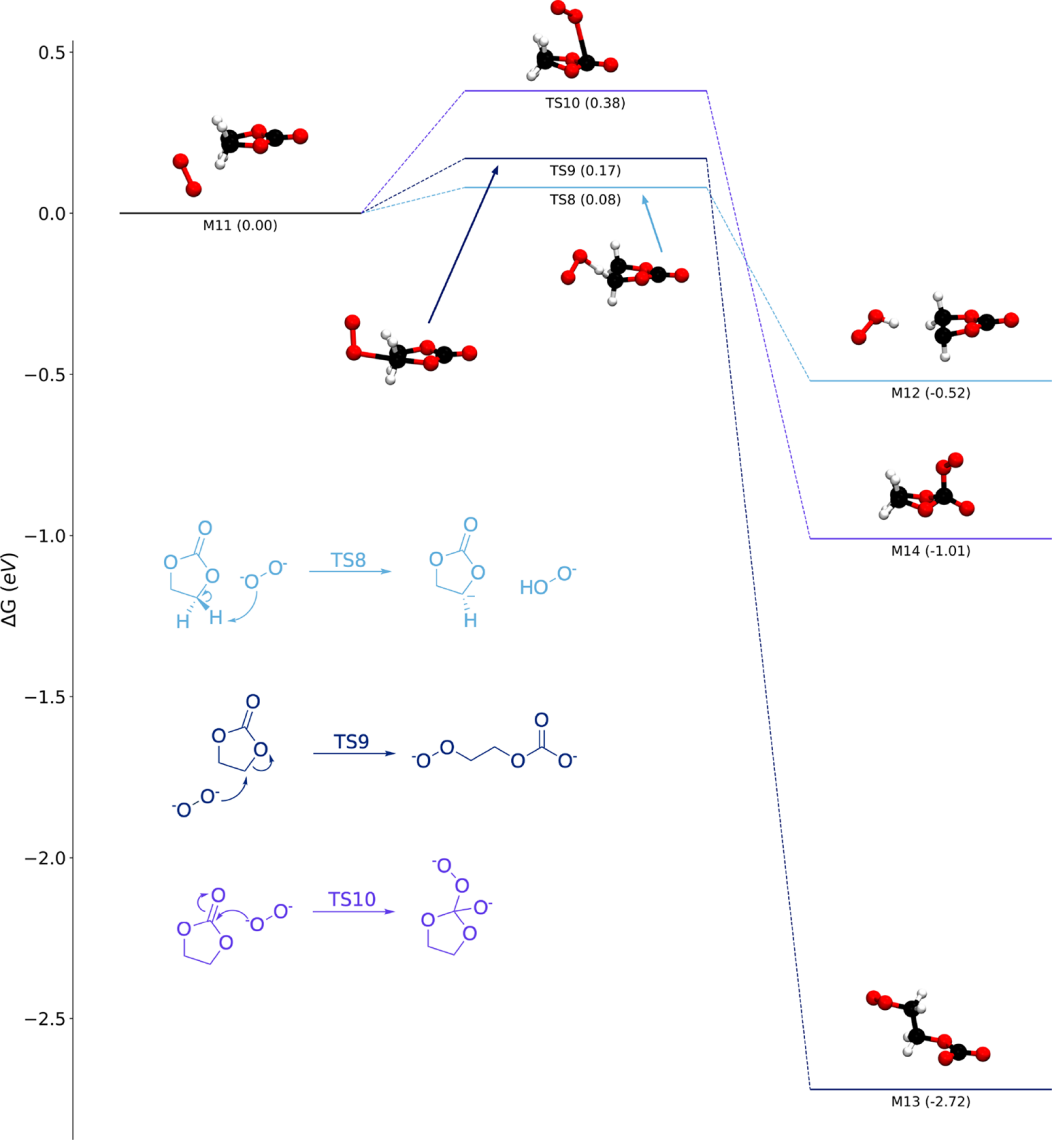
Energy diagrams for reactions between EC and peroxide (O_2_^2–^): proton abstraction (light blue), nucleophilic substitution (dark blue), and addition to form a tetrahedral complex (purple).

We find that O_2_^–^ cannot abstract protons or hydrogen atoms from EC to form H_2_O_2_. The first hydrogen abstraction (M_7_ → M_8_) suffers from a high free energy barrier (Δ*G*^⧧^ = 1.70 eV) and is thermodynamically unfavorable (Δ*G* = 1.62 eV). The removal of an additional proton to form H_2_O_2_ and reduced radical VC is also somewhat unfavorable (M_8_ → M_9_; Δ*G* = 0.08 eV) but has a modest barrier of 0.11 eV.

While O_2_^–^ may not be able to attack EC’s protons, it can attack the ethylene carbons, in agreement with previous studies focused on Li–O_2_ batteries.^51^ We identified a nucleophilic substitution reaction (M_7_ → M_10_). This reaction is favorable (Δ*G* = −0.31 eV), and while it is not predicted to be rapid (Δ*G*^⧧^ = 0.77 eV), it is considerably faster than any ^1^O_2_ reaction that we have identified. For comparison, if we consider the same conditions as we did previously (O_2_^–^ at the saturation concentration of O_2_ dissolved in pure EC at room temperature), the initial rate for the M_7_ → M_10_ reaction (rate_0_) is predicted to be 0.014 M s^–1^, more than 20 orders of magnitude faster than the M_1_ → M_2_ reaction. Aside from the predicted energy barrier, the M_7_ → M_10_ reaction appears to be plausible on the basis of previous experimental observations. Specifically, Kaufman and McCloskey recently used differential electrochemical mass spectroscopy to detect electrolyte decomposition products that formed at lithium-excess NMC electrodes;^21^ they observed peroxide-containing products related to M_10_ and even hypothesized that such species could form via nucleophilic substitution.

Reactions with peroxide are even more facile. Whereas proton abstraction is unfavorable for superoxide, it is predicted to occur rapidly for peroxide (M_11_ → M_12_; Δ*G*^⧧^ = 0.08 eV). Similarly, nucleophilic attack on the ethylene carbons of EC by O_2_^2–^ is extremely favorable and rapid (M_11_ → M_13_; Δ*G* = −2.72 eV; Δ*G*^⧧^ = 0.17 eV). Finally, we predict that peroxide can attach to the carbonate carbon of EC, forming a tetrahedral complex (M_11_ → M_14_; Δ*G*^⧧^ = 0.38 eV).

Here, we have used first-principles DFT calculations to examine common explanations for high-potential electrolyte degradation in LIBs, focusing on model EC-based electrolytes. Even after accounting for the effect of concentration, we found that purely electrochemical oxidation of EC is thermodynamically disfavored at essentially any potential relevant to normal LIB operation. We likewise cast doubt on hypotheses related to ^1^O_2_, as both of the major reactions reported in the literature are predicted to have large kinetic barriers, making them sluggish under ambient conditions.

On the basis of previous studies of oxygen redox in LIB positive electrodes, we suggest the possibility that EC may react with oxygen anions instead of neutral ^1^O_2_. These anionic species are likely to be formed on transition metal oxide surfaces as an intermediate prior to neutral release of the O_2_. Our initial calculations show that EC can react rapidly with O_2_^–^ and especially O_2_^2–^. Additional studies, both computational and experimental, should now be undertaken to assess (i) how easily these anion species can form on NMC, DRX, or other oxide electrode surfaces, (ii) the lifetime of these anions, and (iii) if they can be eliminated from the electrode surface to react homogeneously with the electrolyte, as we have assumed here. We emphasize that in this work we have considered only the initial reactions between EC and oxygen anions. Even if they do occur, we do not yet know if these reactions can lead to observed products, such as water and acids, or what other products and intermediates may be formed. In the future, we intend to use CRN-based methods to more thoroughly explore reactions between EC and oxygen anions.

In this study, we largely ignored the role of electrode surfaces, treating the electrode mainly as a sink for electrons or as a source of various “reactive oxygen” species. However, electrode active material may also directly participate in reactions with electrolyte components such as EC or may act as catalysts. For instance, it has been suggested that EC is dehydrogenated on transition metal oxides.^27,34,52^ While some initial studies of electrolyte reactions with oxide positive electrodes have been conducted using DFT,^53−55^ they have typically been limited to examining a small number of reactions or performing very short dynamic simulations on the picosecond time scale. We hope that further computational studies consider the possible reactive and catalytic nature of LIB positive electrodes in more detail.

We likewise largely ignored salts such as LiPF_6_, choosing to focus on solvent reactivity. Salt anions may also react at increased potentials,^56,57^ and it has been suggested that EC and PF_6_^–^ react together at LIB positive electrodes, forming products such as HF.^58,59^ A complete understanding of LIB electrolyte reactivity at positive electrodes will require additional studies of reaction mechanisms, including salt molecules.

Finally, for the purposes of simplicity and computational cost, we have mostly neglected the role of explicit solvation shells in this work. As strong solvent effects have been identified for particular electrolyte decomposition reactions,^60^ we cannot discount the possibility that the reactions reported here may be accelerated by particular solvent environments. Calculations involving complete solvent clusters should be performed to determine if or how explicit solvation affects reactions between EC and ^1^O_2_, O_2_^–^, or O_2_^2–^.

## Supporting Information Available

Software availability, data availability, computational methods, calculation of the solubility of O_2_ in EC, and cited refs (61−72) (PDF)
Transparent Peer Review report available (PDF)
