# Supplementary material for: A Critical Analysis of Chemical and Electrochemical Oxidation Mechanisms in Li-Ion Batteries
Source: J Phys Chem Lett. 2024 Jan 4;15(2):391–400. doi: 10.1021/acs.jpclett.3c03279 (PMC10801690; doi:10.1021/acs.jpclett.3c03279)
Supplement: Supplementary file 1 — jz3c03279_si_001.pdf [file jz3c03279_si_001.pdf]

Supporting Information: A Critical Analysis of Chemical and Electrochemical Oxidation Mechanisms in Li-Ion Batteries

Evan Walter Clark Spotte-Smith,[†,‡,#] Sudarshan Vijay,[†,¶,#] Thea Bee Petrocelli,[†] Bernardine L. D. Rinkel,[§,‖] Bryan D. McCloskey,[§,‖] and Kristin A. Persson[*,†,⊥]

†*Department of Materials Science and Engineering, University of California – Berkeley, 210 Hearst Memorial Mining Building, Berkeley, CA, 94720 USA*

‡*Materials Science Division, Lawrence Berkeley National Laboratory, 1 Cyclotron Road, Berkeley, CA, 94720 USA*

¶*Present address: VASP Software GmbH, Sensengasse 8, A-1090 Vienna, Austria*

§*Department of Chemical and Biomolecular Engineering, University of California – Berkeley, 201 Gilman Hall, Berkeley, CA, 94720 USA*

‖*Energy Storage and Distributed Resources, Lawrence Berkeley National Laboratory, 1 Cyclotron Road, Berkeley, CA, 94720 USA*

⊥*Molecular Foundry, Lawrence Berkeley National Laboratory, 1 Cyclotron Road, Berkeley, CA, 94720 USA*

#*These authors contributed equally to this work*

E-mail: kapersson@lbl.gov

Software Availability

No software was produced as part of this study.

Data Availability

Data for the isolated molecules used to calculate E^0 and ΔG for electrochemical oxidation reactions are provided on the Materials Project through the Molecules Explorer and associated application programming interface.[1] The unique identifiers (`MPculeIDs`) for the relevant species are given below:

- EC: 176ba51f33c41703bc7ae8d746d124cd-C3H4O3-0-1
- EC^+: 176ba51f33c41703bc7ae8d746d124cd-C3H4O3-1-2
- $(EC{+}H)^+$: f298146429d1e389d786d9bba1e2f0bc-C3H5O3-1-1
- $(EC-H)$: bb526d16d03da9b68c18ed4d87d4d1ef-C3H3O3-0-2

Data for molecular clusters and molecules in energy diagrams are distributed as a JavaScript Object Notation (JSON)-formatted file `ec_ox_o2_data.json` on Figshare (DOI: 10.6084/m9.figshare.24589056).[2] `ec_ox_o2_data.json` contains the structures (as serialized Pymatgen[3] `Molecule` objects) and thermochemical properties of the reaction endpoints and TS reported in this work. For species that appear in energy diagrams (Figures 2–4 in the main text), the corresponding key in `ec_ox_o2_data.json` is the name of the species as reported in the main text. For instance, the data for TS_2 would be found under the key "TS2". EC_2 is listed as "EC2", and the oxidation product cluster $(EC{+}H)^+ + (EC-H)$ is listed as "EC2oxproduct".

To load this data in Python, use monty (https://github.com/materialsvirtuallab/monty):

```
from monty.serialization import loadfn
data = loadfn("ec_ox_o2_data.json")
```

Computational Methods

Molecular density functional theory

Molecular density functional theory (DFT) calculations were performed using version 5 of the Q-Chem electronic structure code.[4] All calculations were performed using the ωB97X-V range-separated hybrid generalized gradient approximation density functional[5] and the def2-TZVPPD basis set.[6] Solvent effects were treated implicitly using the solvent model with density (SMD),[7] using parameters relevant to ethylene carbonate/ethyl methyl carbonate electrolytes that we have reported previously.[8]

All transition-states (TS) were confirmed to have one imaginary frequency and to connect to the expected endpoints. Ground-state minima were confirmed to have zero imaginary frequencies. For clusters, which can often optimize to saddle points with frustrated rotation modes, we allow one imaginary frequency with very small magnitude ($|\nu_i| < 50cm^{-1}$).

Correcting Singlet Oxygen

DFT methods are known to fail pathologically when treating diatomic oxygen in the singlet excited state ($^1O_2$). This was recently demonstrated by Mullinax, Bauschlicher, and Lawson,[9] who used DFT and multireference methods such as complete active space second-order perturbation theory (CASPT2) to calculate the energy barriers of reactions between ($^1O_2$) and small organic molecules. They found that while DFT (using e.g. the M11 range-separated hybrid meta-GGA density functional with the 6-311+G(2d,p) basis set) could capture trends in energy barriers and even relative barriers, DFT severely underpredicts absolute reaction energy barriers. To confirm that this pathological underprediction affected our chosen level of theory (ωB97X-V/def2-TZVPPD), we re-optimized the transition-state and minima structures from the Mullinax-Bauschlicher-Lawson benchmark set. The comparison between CASPT2 and DFT energy barriers is shown in Figure S1.

We hypothesized that the inaccurate DFT barriers were primarily caused by the treat-

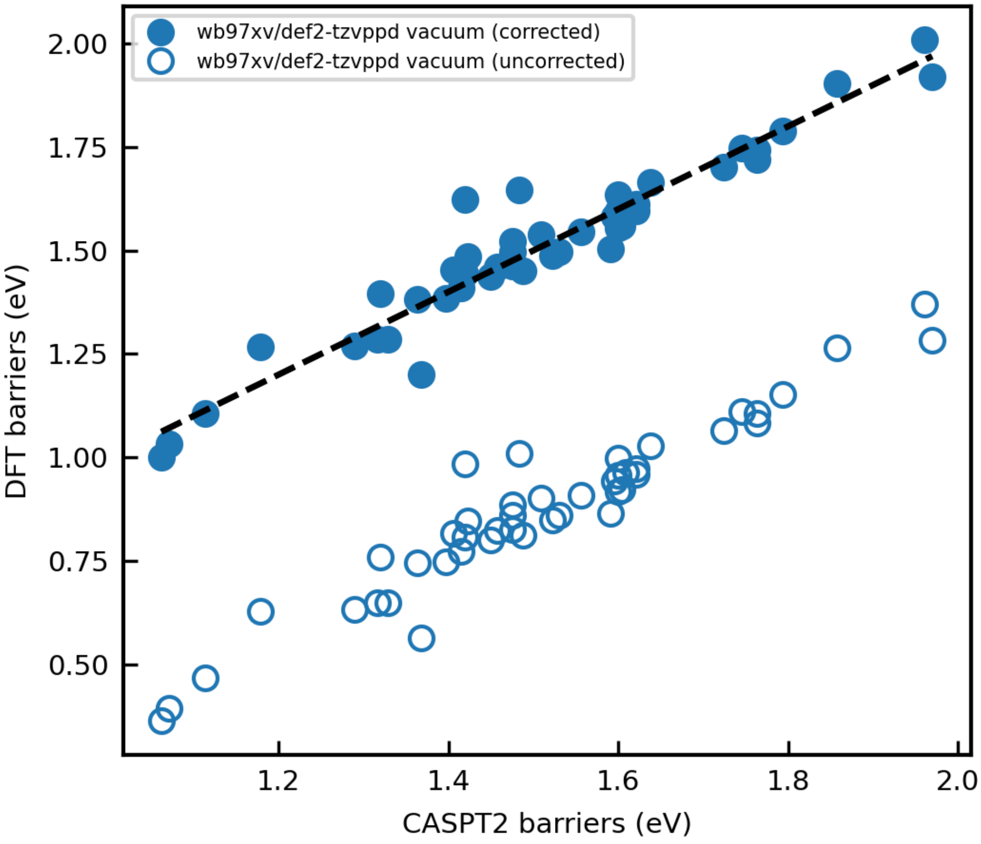

Figure S1: Comparison of reaction barriers computed using the methods used in this manuscript against those computed using the CASPT2 method employed by Mullinax, Bauschlicher, and Lawson.[9]

ment of semi-isolated $^1O_2$, rather than the transition-state. We therefore applied a correction to the reactants in each reaction but did not alter the thermochemistry of the transition-states or reaction products. Specifically, we lowered the electronic energy of the reactant by 0.645 eV, corresponding to the difference between the experimental singlet-triplet gap of O_2 (0.977 eV)[10] and the DFT-calculated gap (1.622 eV at the ωB97X-V/def2-TZVPPD/vacuum level of theory). As Figure S1 shows, with this correction applied, the DFT-calculated energy barriers agree well with those calculated via CASPT2.

Although we considered superoxide (${O_2}^{-1}$) and peroxide (${O_2}^{-2}$) only in their ground states (doublet and singlet, respectively), it is nonetheless worth considering if a similar correction should be applied to these oxygen anions. We do not believe that correcting ${O_2}^{-1}$ or ${O_2}^{-2}$ is necessary based on comparison with available reference data. For instance, our chosen level of theory is able predicts the gas-phase adiabatic electron affinity of $^3O_2$ ($O_2 + e^- \longrightarrow {O_2}^{-1}$) in reasonable agreement with experiment (0.348 eV calculated vs. 0.448 eV experimental).[11]

Electrochemistry

We calculate standard adiabatic oxidation potentials based on the Gibbs free energy (ΔG) of the reaction $A \longrightarrow A^+ + e^-$. Specifically,

$$E^\circ(\mathrm{Li/Li^+}) = ({G_{A^+}}^\circ - {G_A}^\circ) - 1.4\mathrm{V} \tag{1}$$

where ${G_i}^\circ$ is the standard-state Gibbs free energy of species i and the shift by 1.4 V is necessary to convert to a Li/Li$^+$ reference.

Solubility of O_2 in EC

The Bunsen coefficient (α) is a measure of the volume of a gas at 273.15 K and 1 atm (101,325 Pa) that is absorbed per unit volume of some liquid solution under 1 atm pressure. Read

et al.[12] previously calculated the Bunsen coefficient of O_2 in EC based on measurements of mixtures of EC and propylene carbonate (PC). Using a pure O_2 atmosphere at 298.15 K, they obtained $\alpha_{O_2/EC} = 0.0382$. In other words, 38.2 mL of gaseous O_2 can be dissolved in 1 L of EC under these conditions.

If we treat O_2 as an ideal gas, then the ideal gas law states that 38.2 mL of O_2 at 298.15 K and 1 atm is 1.56 millimoles of O_2, which means that the maximum concentration of O_2 in EC is 1.56 mM.

References

(1) Spotte-Smith, E. W. C.; Cohen, O. A.; Blau, S. M.; Munro, J. M.; Yang, R.; Guha, R. D.; Patel, H. D.; Vijay, S.; Huck, P.; Kingsbury, R.; Horton, M. K.; Persson, K. A. A Database of Molecular Properties Integrated in the Materials Project. *Digital Discovery* **2023**, *2*, 1862–1882.

(2) Spotte-Smith, E. W. C.; Vijay, S.; Petrocelli, T. B.; Rinkel, B. L. D.; McCloskey, B. D.; Persson, K. A. Data for "A Critical Analysis of Chemical and Electrochemical Oxidation Mechanisms in Li-Ion Batteries". 2023; Figshare. https://doi.org/10.6084/m9.figshare.24589056 (accessed Dec. 18, 2023).

(3) Ong, S. P.; Richards, W. D.; Jain, A.; Hautier, G.; Kocher, M.; Cholia, S.; Gunter, D.; Chevrier, V. L.; Persson, K. A.; Ceder, G. Python Materials Genomics (Pymatgen): A Robust, Open-Source Python Library for Materials Analysis. *Computational Materials Science* **2013**, *68*, 314–319.

(4) Epifanovsky, E.; Gilbert, A. T. B.; Feng, X.; Lee, J.; Mao, Y.; Mardirossian, N.; Pokhilko, P.; White, A. F.; Coons, M. P.; Dempwolff, A. L.; Gan, Z.; Hait, D.; Horn, P. R.; Jacobson, L. D.; Kaliman, I.; Kussmann, J.; Lange, A. W.; Lao, K. U.; Levine, D. S.; Liu, J.; McKenzie, S. C.; Morrison, A. F.; Nanda, K. D.; Plasser, F.;

Rehn, D. R.; Vidal, M. L.; You, Z.-Q.; Zhu, Y.; Alam, B.; Albrecht, B. J.; Aldossary, A.; Alguire, E.; Andersen, J. H.; Athavale, V.; Barton, D.; Begam, K.; Behn, A.; Bellonzi, N.; Bernard, Y. A.; Berquist, E. J.; Burton, H. G. A.; Carreras, A.; Carter-Fenk, K.; Chakraborty, R.; Chien, A. D.; Closser, K. D.; Cofer-Shabica, V.; Dasgupta, S.; de Wergifosse, M.; Deng, J.; Diedenhofen, M.; Do, H.; Ehlert, S.; Fang, P.-T.; Fatehi, S.; Feng, Q.; Friedhoff, T.; Gayvert, J.; Ge, Q.; Gidofalvi, G.; Goldey, M.; Gomes, J.; González-Espinoza, C. E.; Gulania, S.; Gunina, A. O.; Hanson-Heine, M. W. D.; Harbach, P. H. P.; Hauser, A.; Herbst, M. F.; Hernández Vera, M.; Hodecker, M.; Holden, Z. C.; Houck, S.; Huang, X.; Hui, K.; Huynh, B. C.; Ivanov, M.; Jász, ; Ji, H.; Jiang, H.; Kaduk, B.; Kähler, S.; Khistyaev, K.; Kim, J.; Kis, G.; Klunzinger, P.; Koczor-Benda, Z.; Koh, J. H.; Kosenkov, D.; Koulias, L.; Kowalczyk, T.; Krauter, C. M.; Kue, K.; Kunitsa, A.; Kus, T.; Ladjánszki, I.; Landau, A.; Lawler, K. V.; Lefrancois, D.; Lehtola, S.; Li, R. R.; Li, Y.-P.; Liang, J.; Liebenthal, M.; Lin, H.-H.; Lin, Y.-S.; Liu, F.; Liu, K.-Y.; Loipersberger, M.; Luenser, A.; Manjanath, A.; Manohar, P.; Mansoor, E.; Manzer, S. F.; Mao, S.-P.; Marenich, A. V.; Markovich, T.; Mason, S.; Maurer, S. A.; McLaughlin, P. F.; Menger, M. F. S. J.; Mewes, J.-M.; Mewes, S. A.; Morgante, P.; Mullinax, J. W.; Oosterbaan, K. J.; Paran, G.; Paul, A. C.; Paul, S. K.; Pavošević, F.; Pei, Z.; Prager, S.; Proynov, E. I.; Rák, ; Ramos-Cordoba, E.; Rana, B.; Rask, A. E.; Rettig, A.; Richard, R. M.; Rob, F.; Rossomme, E.; Scheele, T.; Scheurer, M.; Schneider, M.; Sergueev, N.; Sharada, S. M.; Skomorowski, W.; Small, D. W.; Stein, C. J.; Su, Y.-C.; Sundstrom, E. J.; Tao, Z.; Thirman, J.; Tornai, G. J.; Tsuchimochi, T.; Tubman, N. M.; Veccham, S. P.; Vydrov, O.; Wenzel, J.; Witte, J.; Yamada, A.; Yao, K.; Yeganeh, S.; Yost, S. R.; Zech, A.; Zhang, I. Y.; Zhang, X.; Zhang, Y.; Zuev, D.; Aspuru-Guzik, A.; Bell, A. T.; Besley, N. A.; Bravaya, K. B.; Brooks, B. R.; Casanova, D.; Chai, J.-D.; Coriani, S.; Cramer, C. J.; Cserey, G.; DePrince, A. E., III; DiStasio, R. A., Jr.; Dreuw, A.; Dunietz, B. D.; Furlani, T. R.; Goddard, W. A., III; Hammes-Schiffer, S.; Head-

Gordon, T.; Hehre, W. J.; Hsu, C.-P.; Jagau, T.-C.; Jung, Y.; Klamt, A.; Kong, J.; Lambrecht, D. S.; Liang, W.; Mayhall, N. J.; McCurdy, C. W.; Neaton, J. B.; Ochsenfeld, C.; Parkhill, J. A.; Peverati, R.; Rassolov, V. A.; Shao, Y.; Slipchenko, L. V.; Stauch, T.; Steele, R. P.; Subotnik, J. E.; Thom, A. J. W.; Tkatchenko, A.; Truhlar, D. G.; Van Voorhis, T.; Wesolowski, T. A.; Whaley, K. B.; Woodcock, H. L., III; Zimmerman, P. M.; Faraji, S.; Gill, P. M. W.; Head-Gordon, M.; Herbert, J. M.; Krylov, A. I. Software for the Frontiers of Quantum Chemistry: An Overview of Qevelopments in the Q-Chem 5 Package. *The Journal of Chemical Physics* **2021**, *155*, 084801.

(5) Mardirossian, N.; Head-Gordon, M. B97X-V: A 10-parameter, Range-Separated Hybrid, Generalized Gradient Approximation Density Functional with Nonlocal Correlation, Designed by a Survival-of-the-Fittest Strategy. *Physical Chemistry Chemical Physics* **2014**, *16*, 9904–9924.

(6) Rappoport, D.; Furche, F. Property-Optimized Gaussian Basis Sets for Molecular Response Calculations. *The Journal of Chemical Physics* **2010**, *133*, 134105.

(7) Marenich, A. V.; Cramer, C. J.; Truhlar, D. G. Universal Solvation Model Based on Solute Electron Density and on a Continuum Model of the Solvent Defined by the Bulk Dielectric Constant and Atomic Surface Tensions. *The Journal of Physical Chemistry B* **2009**, *113*, 6378–6396.

(8) Spotte-Smith, E. W. C.; Blau, S. M.; Xie, X.; Patel, H. D.; Wen, M.; Wood, B.; Dwaraknath, S.; Persson, K. A. Quantum Chemical Calculations of Lithium-Ion Battery Electrolyte and Interphase Species. *Scientific Data* **2021**, *8*, 203.

(9) Mullinax, J. W.; Bauschlicher, C. W. J.; Lawson, J. W. Reaction of Singlet Oxygen with the Ethylene Group: Implications for Electrolyte Stability in Li-Ion and Li-O2 Batteries. *The Journal of Physical Chemistry A* **2021**, *125*, 2876–2884.

(10) Schweitzer, C.; Schmidt, R. Physical Mechanisms of Generation and Deactivation of Singlet Oxygen. *Chemical Reviews* **2003**, *103*, 1685–1758.

(11) Ervin, K. M.; Anusiewicz, I.; Skurski, P.; Simons, J.; Lineberger, W. C. The Only Stable State of O_2^- Is the $X^2{}_g$ Ground State and It (Still!) Has an Adiabatic Electron Detachment Energy of 0.45 eV. *The Journal of Physical Chemistry A* **2003**, *107*, 8521–8529.

(12) Read, J.; Mutolo, K.; Ervin, M.; Behl, W.; Wolfenstine, J.; Driedger, A.; Foster, D. Oxygen Transport Properties of Organic Electrolytes and Performance of Lithium/Oxygen Battery. *Journal of the Electrochemical Society* **2003**, *150*, A1351.
