# Supplementary material for: A Critical Analysis of Chemical and Electrochemical Oxidation Mechanisms in Li-Ion Batteries
Source: J Phys Chem Lett. 2024 Jan 4;15(2):391–400. doi: 10.1021/acs.jpclett.3c03279 (PMC10801690; doi:10.1021/acs.jpclett.3c03279)
Supplement: Supplementary file 2 — jz3c03279_si_002.pdf [file jz3c03279_si_002.pdf]

jz-2023-03279f.R1

Name: Peer Review Information for "A Critical Analysis of Chemical and Electrochemical Oxidation Mechanisms in Li-Ion Batteries"

First Round of Reviewer Comments

Reviewer: 1

Comments to the Author

This is an excellent contribution to the electrochemical energy storage field. The manuscript should be accepted with the following minor revisions. On page 8 and 9, the authors refer to 1,3-dioxolane-2,4-dione as a diketone. This is not a diketone, but could be called a dicarbonyl. Figure 2 would look better if the colors were significantly different. It is difficult to distinguish light orange from dark orange. On page 15, the authors mention that they have largely ignored the effect of electrode surfaces. They should also mention that they have largely ignored the effect of the salt. PF6- has been reported to be important in EC oxidation. ACS Energy Lett. 2021, 6, 3788−3792 and J. Electrochem. Soc. 2018, 165 (13), A3022−A3028.

Author's Response to Peer Review Comments:

Dear Prof. Editor,

We have received your e-mail containing the review for "A Critical Analysis of Chemical and Electrochemical Oxidation Mechanisms in Li-ion Batteries" (manuscript ID jz-2023-03279f) by Spotte-Smith, Vijay, et al. submitted to *The Journal of Physical Chemistry Letters*. We would like to thank the reviewer for their assessment of the paper and their feedback. The reviewer's comments and our responses, along with the changes made, are described below.

Reviewer #1:

1. **Comment**: This is an excellent contribution to the electrochemical energy storage field.

Response: We thank the reviewer for this kind comment!

Changes: None

2. **Comment**: On page 8 and 9, the authors refer to 1,3-dioxolane-2,4-dione as a diketone. This is not a diketone, but could be called a dicarbonyl.

Response: We appreciate the reviewer pointing out this issue in nomenclature. We have removed the use of the term “diketone” and replaced it with “dicarbonyl”.

Changes:

“In the first (orange) pathway, originally suggested by Jung et al., $^1O2$ initially reacts with EC to form water and

1,3-dioxolane-2,4-dione, a dicarbonyl species.”

“Figure 2: Energy diagrams for two reactions between $^1O2$ and EC: one in which $^1O2$ eventually forms H2O and a dicarbonyl species (orange) and one in which $^1O2$ abstracts two hydrogen atoms from EC to form H2O2 and vinylene carbonate (VC) in a single concerted step (yellow).”

3. **Comment**: Figure 2 would look better if the colors were significantly different. It is difficult to distinguish light orange from dark orange.

Response: We have changed the color scheme of Figure 2 to aid readability.

Changes:

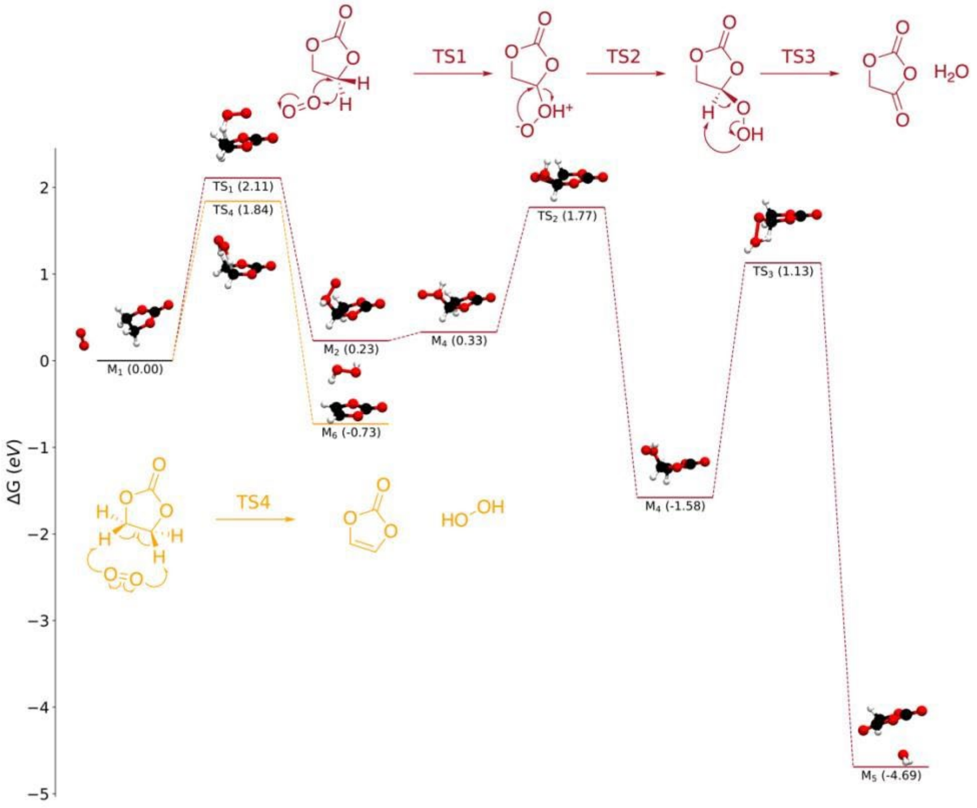

4. **Comment**: On page 15, the authors mention that they have largely ignored the effect of electrode surfaces. They should also mention that they have largely ignored the effect of the salt. PF6- has been reported to be important in EC oxidation.

Response: The reviewer brings up an important point. In this paper, we have not studied the decomposition of LiPF6 or $PF6^-$ at Li-ion battery positive electrodes, nor have we considered how EC and LiPF6/$PF6^-$ could react together at elevated potentials. We have now addressed this limitation of our analysis.

Changes: "We have likewise largely ignored salts such as LiPF6, choosing to focus on solvent reactivity. Salt anions may also react at elevated potentials, and it has been suggested that EC and $PF6^-$ react together at LIB positive electrodes, forming products such as HF. A complete understanding of LIB electrolyte reactivity at positive electrodes will require additional studies into reaction mechanisms including salt molecules."

In addition to the changes above, we have responded to the non-scientific changes requested by the editorial staff. We believe that with the responses above and the modifications to the manuscript, we have addressed the reviewer's concerns, and we hope that our manuscript will be accepted for publication as a Letter in *The Journal of Physical Chemistry Letters*.

Sincerely,

Kristin A Persson
Daniel M. Tellep Distinguished Professor of Materials Science and Engineering, University of California at Berkeley

Faculty Senior Scientist, Lawrence Berkeley National Laboratory

Director, Materials Project
(www.materialsproject.org) Director, Molecular
Foundry (foundry.lbl.gov) kapersson@lbl.gov
